# Lower limb edema detection and grading classification using deep learning and image enhancement technologies

**DOI:** 10.3389/fmed.2026.1697503

**Published:** 2026-03-11

**Authors:** Ting-Wei Hu, Chao-Hung Wang, Min-Hui Liu, Hui-Huang Hsu, Tun-Wen Pai

**Affiliations:** 1 Programs of Artificial Intelligence Technology, Innovation Frontier Institute of Research for Science and Technology, National Taipei University of Technology, Taipei, Taiwan; 2 Heart Failure Research Center, Division of Cardiology, Department of Internal Medicine, Chang Gung Memorial Hospital, Keelung, Taiwan; 3 School of Medicine, Chang Gung University, Taoyuan, Taiwan; 4 Department of Computer Science and Information Engineering, Tamkang University, New Taipei City, Taiwan; 5 Department of Computer Science and Information Engineering, National Taipei University of Technology, Taipei, Taiwan

**Keywords:** deep learning, edema grading, image enhancement, lower limb edema, YOLO

## Abstract

**Background:**

Lower limb edema is a common clinical symptom closely associated with chronic diseases such as heart failure, liver disease, and renal dysfunction. Edema severity grading is an important indicator for clinical diagnosis and disease monitoring. However, traditional assessments that rely on visual inspection and manual palpation are subjective and inconsistent, making them insufficient to meet the requirements of standardized and precise diagnostics.

**Methods:**

This study proposes a multistage deep learning framework that integrates object detection and image classification for automatic detection and grading of lower limb edema. The system architecture initially employed YOLO models to detect indentation regions, followed by image enhancement techniques to improve the representation of edema features and enhance the detection accuracy. Finally, classification models were used to categorize edema severity. To address the data imbalance issue, random rotation was applied for data augmentation, and non-target regions were removed through automatic background elimination and cropping to enhance classification performance.

**Results:**

The experimental findings demonstrated that the proposed system achieved an average classification accuracy of 87~93% across different edema severity levels, 90–94% for recall rates, and 93~97% for precisions for different edema stages. These results validate the feasibility and effectiveness of the automatic detection and grading classification system for lower limb edema.

**Conclusion:**

The proposed system holds the potential for both clinical decision support and home-based self-care, enhancing the accuracy and consistency of edema assessment for patients and healthcare professionals. It can facilitate smart and precision medicine based on the status of lower limb edema.

## 1 Introduction

Lower limb edema is a common clinical symptom, typically resulting from the abnormal accumulation of interstitial fluid, and is frequently associated with chronic conditions, such as heart failure, liver disease, or renal dysfunction (1). Edema severity could serve as a crucial indicator for clinicians for both rapid diagnosis and disease monitoring. Accurate grading of edema severity provides essential insights for the timely evaluation of disease progression and guides subsequent therapeutic decision-making. Therefore, precise assessment of edema grading is not only critical for clinical management but also indispensable for the early detection of disease deterioration.

The conventional assessment of edema severity primarily relies on clinicians who typically employ visual inspection and manual palpation techniques. Although this approach is widely applied in clinical practice, it is inherently subjective and susceptible to inter-observer variability. Specifically, physicians press on the patient’s lower limb skin and evaluate the depth of the indentation and the rebounding time to determine the grade of edema (2). However, when different clinicians examine the same patient, diagnostic outcomes may vary despite observing identical degrees of indentation, underscoring a lack of objectivity and consistency (3). With the increasing complexity of chronic disease management and the rising number of clinical visits, traditional physician-based assessments or patient self-evaluations at home are incapable of meeting the growing demand for standardized and precise diagnostic practices in modern healthcare.

To address the lack of objectivity and consistency commonly observed in traditional lower limb edema assessments, this study proposes a multi-stage deep learning framework that integrates automated object detection and image classification for the automatic detection and grading of lower limb edema. By leveraging artificial intelligence to replace conventional manual evaluation, a standardized diagnostic workflow could be established, which would not only enhance the accuracy of lower limb edema assessment but also substantially reduce errors introduced by manual palpation.

The system architecture is designed as a multistage pipeline. First, the YOLO model was employed to detect the initial locations of edema indentations. Subsequently, image enhancement techniques are applied to highlight edema-related features, thereby improving the accuracy of advanced detection. Finally, deep learning-based classification was integrated to achieve automatic grading of edema severity. In the early stages of this study, lower limb edema images were manually annotated by a senior clinical specialist with over 20 years of experience. The annotations included the locations of indentation following manual pressure and the corresponding severity grades of edema, which served as the ground truth for model training. To address data imbalance, limited samples of higher severity grades of edema were augmented through random rotation. Furthermore, non-target regions within the images were automatically removed and cropped to improve detection accuracy. For model evaluation, k-fold cross-validation was conducted to assess the stability and reliability of the automated recognition results. Furthermore, multiple classification strategies were implemented to analyze and compare the system’s performance in automated edema detection and severity grading.

This study’s primary aim was to develop an objective and accurate method for the automatic detection and grading of lower limb edema. In the near future, this system will be directly applied to both clinical decision support and home-based self-care, thereby enhancing diagnostic accuracy for healthcare professionals and contributing to the broader vision of smart healthcare and precision medicine.

Lower limb edema refers to the abnormal retention of fluid within the interstitial space outside blood vessels, resulting in localized swelling of the affected area. The underlying pathophysiological mechanisms include decreased plasma colloid osmotic pressure, increased capillary hydrostatic pressure, enhanced capillary wall permeability, impaired lymphatic drainage, and abnormalities in the renin–angiotensin–aldosterone system, all of which contribute to fluid and sodium retention within the body (4). These factors may occur independently or interact synergistically, leading to interstitial fluid accumulation that fails to adequately return to the circulatory system, ultimately causing edema.

Regarding advances in medical devices for edema assessment, preliminary self-examinations can be performed using simple physical palpation. A commonly employed method involves gently pressing the calf muscle with the index finger and observing the depth of indentation and rebound time as indicators of edema severity. Although this technique is relatively simple, it is significant and practical for both clinical practice and home-based care.

According to commonly adopted clinical grading standards, edema severity is classified into five levels (0 to $4^{+}$ ), primarily based on the depth of indentation and the rebound time observed following skin compression. Grade 0 indicated the absence of noticeable edema, whereas grades $1^{+}$ to $4^{+}$ corresponded to progressively greater indentation depths and longer rebound times, respectively (5). For example, an indentation depth of approximately 4 mm, with a rebound time ranging from 1~10 s, is generally classified as grade 2 edema. The detailed criteria for the different grades are summarized in Table 1.

**Table 1:** Edema grading criteria.

Grading	Indentation depth	Rebound time
0	0 mm	0 s
$1^{+}$	2 mm	0~1 s
$2^{+}$	4 mm	1~10 s
$3^{+}$	6 mm	10~20 s
$4^{+}$	8 mm	More than 30 s

In this study, the edema grading standard was adopted as a reference for data annotation. Physicians manually labeled the edema categories in the images according to indentation depth, which were subsequently used to train the deep learning model for automatic recognition of different edema grades, thereby forming the basis for subsequent automated classification. Traditionally, the assessment of lower limb edema has relied on manual inspection and palpation-based measurements. However, several alternative approaches have been reported, including pressure-based sensing for plantar load evaluation (6), the interpretation of irregular plantar skin temperature variations (7), and three-dimensional (3D) imaging techniques for quantifying the degree of limb swelling (8). In addition to the usage of sensor devices for edema detection (9), the application of image processing techniques for edema classification (10) and the adoption of deep learning methods for edema grading analysis (11) were also published. More recently, short-wave infrared molecular chemical imaging (SWIR MCI) was also introduced as a non-contact modality for objective edema evaluation (12).

To describe in more details, in the early development of lower limb edema assessment, various monitoring strategies centered on sensor-based technologies have been popularly proposed. One representative approach focused on the analysis of plantar load characteristics. The SurroSense Rx smart footwear system developed by the Canadian startup Orpyx integrated multiple pressure sensors within the insole to collect raw data (6), which were subsequently transformed and analyzed for evaluating abnormalities in plantar pressure distribution, and to provided supportive information for identifying foot pathology and potential edema-related risks for patients. Another type approach emphasized risk assessment based on abnormal variations in plantar skin temperature. The Siren Socks smart textile system was developed by the U.S. startup Siren Care. It embedded multiple miniature temperature sensors within the fabric to enable long-term monitoring of temperature distributions across several high-risk regions of the feet in diabetic patients (7). Because diabetic foot complications were often associated with impaired peripheral circulation, leading to localized swelling, infection, and ulceration. The proposed system could facilitate early intervention by issuing real-time alerts via mobile devices when asymmetric or elevated temperature patterns were detected. Another type of 3D imaging measurement techniques was proposed to quantify the degree of limb swelling. The system was designed by Yahathugoda et al. (8) and applied for edema monitoring in patients after lymph node dissection. Finally, using infrared depth-sensing technology analogous to structured depth-scanning approaches, this system generated accurate virtual 3D reconstructions of the lower extremities, allowing precise estimation of limb volume and circumference, and offering an alternative to conventional tape-measure measurements and invasive drainage-based evaluation methods.

George et al. (9) proposed the HeartSMART system for edema detection, which employs sensors to measure skin indentation depth across different edema grades. In their study, memory foam was used as a surrogate skin sample to simulate various degrees of edema. The system integrates both force and displacement sensors, applies constant pressure to the samples, and records the resulting indentation depths for assessment. However, because of the displacement sensor’s maximum measurable range (9.75 mm), the experiment was limited to evaluating edema up to grade 3. According to clinical data, the maximum indentation depth observed in cases of severe edema is approximately 8 mm, which remains within the operational range of the sensors during patient testing.

Williams et al. (10) introduced a system termed Air Edema Reporting (AERO) for edema detection, which utilizes a high-speed camera to monitor the surface deformations of a surrogate skin model when subjected to compressed air. In this study, the Lifeform® synthetic skin model was employed as the experimental substrate, and edema severity was graded on a scale ranging from 0 to $4^{+}$ . The system was capable of distinguishing different edema grades and providing quantitative data on rebound times. However, the performance of an AERO system is easily affected by low-light or dark conditions. Furthermore, the experimental processes still require manual operation, and the procedural complexity and time consumption pose challenges for ensuring consistency. These limitations highlight the need for further refinement before the system can be applied in clinical practice.

Chen and Mao (11) conducted a study in which 1,732 videos of synthetic skin models with varying degrees of edema were recorded and classified into five grades (from stage 0 to stage 4). Two distinct datasets were constructed to investigate the impact of image preprocessing on model performance. The first dataset consisted of single-frame images, and the second was generated by stacking three images captured at different time points (0, 10, and 20 s). From the designed model, both a traditional machine-learning approach (HOG + SVM) and a deep-learning architecture (AlexNet) were employed. Cross-experiments were conducted by pairing each dataset with the two models to compare their classification accuracies. The results demonstrated that the HOG + SVM model achieved an accuracy of up to 94.2%, whereas AlexNet achieved an accuracy of 98.8%. However, this study was limited by its reliance on synthetic skin models, and its performance in clinical applications remains uncertain.

Smith et al. (12) introduced a non-contact assessment approach for peripheral edema based on short-wave infrared (SWIR) molecular chemical imaging (MCI), focusing on the anterior tibial region in both healthy individuals and patients with heart failure. In their study, a total of 103 shin samples were analyzed, and the spectral findings indicated that edematous tissue exhibited increased water-related absorption characteristics within the SWIR range. Using partial least squares discriminant analysis (PLS-DA), the proposed method achieved an accuracy of 97.1% in distinguishing healthy subjects from those presenting various grades of pitting edema. Furthermore, partial least squares regression (PLSR) was employed to estimate edema severity, yielding accuracy of 81.6% across edema grades. These results demonstrated that SWIR MCI could provide reliable differentiation and grading performance even with a relatively limited sample size, highlighting its potential as an objective tool for peripheral edema evaluation.

In recent years, with the rapid advancement of artificial intelligence technologies, machine learning and deep learning methods have been widely applied across various medical domains, including disease prediction (13), clinical decision support (14), and medical image analysis (15), thereby providing new opportunities and directions for medical research.

Among these studies, Yang et al. (16) proposed a convolutional neural network–based framework for the detection and classification of scaphoid fractures in radiographic images. Their model adopted a two-stage architecture, in which Faster R-CNN was firstly employed to localize the scaphoid bone, followed by fracture classification using a ResNet backbone integrated with a feature pyramid network (FPN) and a multi-view fusion module, resulting in improved classification accuracy. Tarimo et al. (17) introduced a method that combined object detection and image classification for polymorphonuclear leukocyte analysis, utilizing a hybrid architecture based on YOLO and Vision Transformer (ViT), which demonstrated superior performance in classification tasks. In addition, Elazab et al. (18) also developed a hybrid deep learning model that integrated YOLOv5 and ResNet50 for tumor localization and classification in histopathological images, achieving effective identification of brain tumors from tissue pathology data.

Motivated by these published articles and to address the limitations of conventional lower limb edema assessment methods, particularly for the high subjectivity and limited inter-rater consistency, this study proposes a multi-stage deep learning framework that combines object detection and image classification for automatic detection and providing the severity grades of lower limb edema.

## 2 Materials and methods

### 2.1 Overall architecture

**Figure 1:**
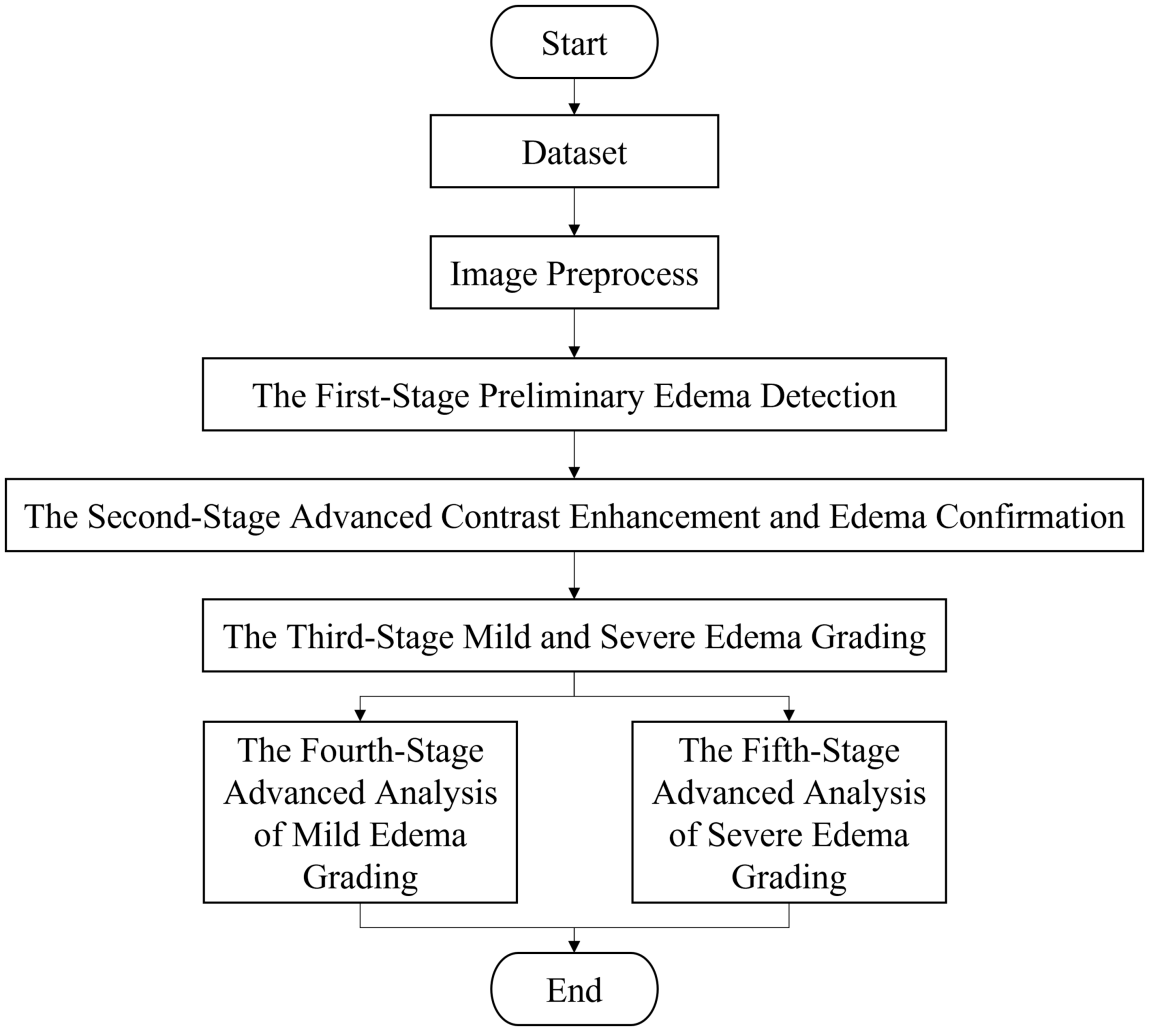
System architecture flowchart.

This paper proposes a system for the automatic detection and grading of edema in real lower-limb skin images. To achieve a more precise classification of edema severity, a hierarchical classification strategy was designed, consisting of an initial coarse classification followed by a fine-grained subclassification. The overall workflow of the system is divided into five major stages, as illustrated in Figure 1. These include: (1) the first stage preliminary edema detection, (2) the second stage advanced contrast enhancement and edema confirmation, (3) the third stage of mild and severe edema grading, (4) the fourth stage advanced analysis of mild edema grading, and (5) the fifth stage advanced analysis of severe edema grading. The specific functions and processes at each stage are described in detail below.

At the first stage, termed “Preliminary Edema Detection,” a lower limb edema image dataset was established. A senior clinical specialist with more than 20 years of experience manually annotated and classified the indentation regions of edema within the images. The dataset was then subjected to preprocessing, which included background removal, image cropping, and data augmentation through image rotation to address class imbalance and enhance the feature-learning capacity. Subsequently, all images were resized to 640 × 640 pixels to meet the input requirements of the YOLO framework. YOLO-based object detection algorithms, including YOLOv9 (19), YOLOv10 (20), and YOLOv11 (21), were employed for training the primary models used in the initial stage of edema detection. Based on the evaluation metrics obtained from model training, the detection model with the best performance was selected as the basis for proceeding to the second stage of the workflow.

The second stage, termed “Advanced Contrast Enhancement and Edema Confirmation,” was designed to improve the accuracy of edema detection by re-evaluating the outcomes of the preliminary detection stage. For images initially identified as non-edematous, three contrast enhancement techniques, including histogram equalization (HE), adaptive histogram equalization (AHE), and contrast-limited adaptive histogram equalization (CLAHE), were applied to enhance feature visibility. The enhanced images were subsequently reanalyzed, and the best-performing model was used for secondary model training. Finally, based on the detection results obtained from the three different contrast enhancement techniques, the best-performing detection model was selected. Furthermore, for edema region detection during the first stage, the template-matching technique in OpenCV was employed to align the pre- and post-compressed images, identifying consistent indentation patterns. This process enables the identification of previously missed or misclassified cases, thereby reducing the number of false positives and false negatives. Ultimately, only rigorously confirmed edema-positive images were retained, serving as the foundation for subsequent grading stages.

For the third stage, termed “Mild and Severe Edema Grading,” categorized edema severity into two groups based on clinical standards: mild edema (Edema $\;1^{+}2^{+}$ ) and severe edema (Edema $\;3^{+}4^{+}$ ). At this stage, images confirmed in the second stage to contain edema indentation regions were firstly extracted and resized to 224 × 224 pixels as input for the classification model. These images, annotated with edema severity levels, were then applied to severity grading using a deep learning–based classification model, enabling the model to effectively learn the characteristic differences between mild and severe edema levels. Several state-of-the-art deep learning models were employed for training and evaluation, including VGG16 (22), ResNet50 (23), YOLOv8 (24), and YOLOv11 (21). Based on the evaluation metrics, the best-performing model was selected to serve as the foundation for subsequent stages dedicated to the detailed analysis of the mild and severe edema groups.

The fourth stage, termed “Advanced Analysis of Mild Edema Grading,” focused on images classified in Stage 3 as mild edema (Edema $\;1^{+}2^{+}$ ) and further refined the automatic classification into Edema $\;1^{+}$ and Edema $\;2^{+}$ . The primary objective of this stage is to differentiate between varying levels of mild edema, thereby facilitating the early detection of subtle changes and enhancing their clinical utility. Deep learning models, including VGG16 (22), ResNet50 (23), YOLOv8 (24), and YOLOv11 (21), were individually employed to train and evaluate the detailed feature variations presented in mild edema images.

The fifth stage, termed “Advanced Analysis of Severe Edema Grading,” focused on images classified in Stage 3 as severe edema (Edema $\;3^{+}4^{+}$ ) and further subdivided them into Edema $\;3^{+}$ and Edema $\;4^{+}$ , respectively. The primary objective of this stage was to differentiate between the varying degrees of severe edema by analyzing the depth and morphological features, thereby providing insights into the progression of advanced edema. Deep learning models, such as the previous modules, were employed for training and evaluating subtle feature variations in severe edema images to ensure accurate subclassification of advanced edema severity.

### 2.2 Dataset and preprocessing

**Figure 2:**
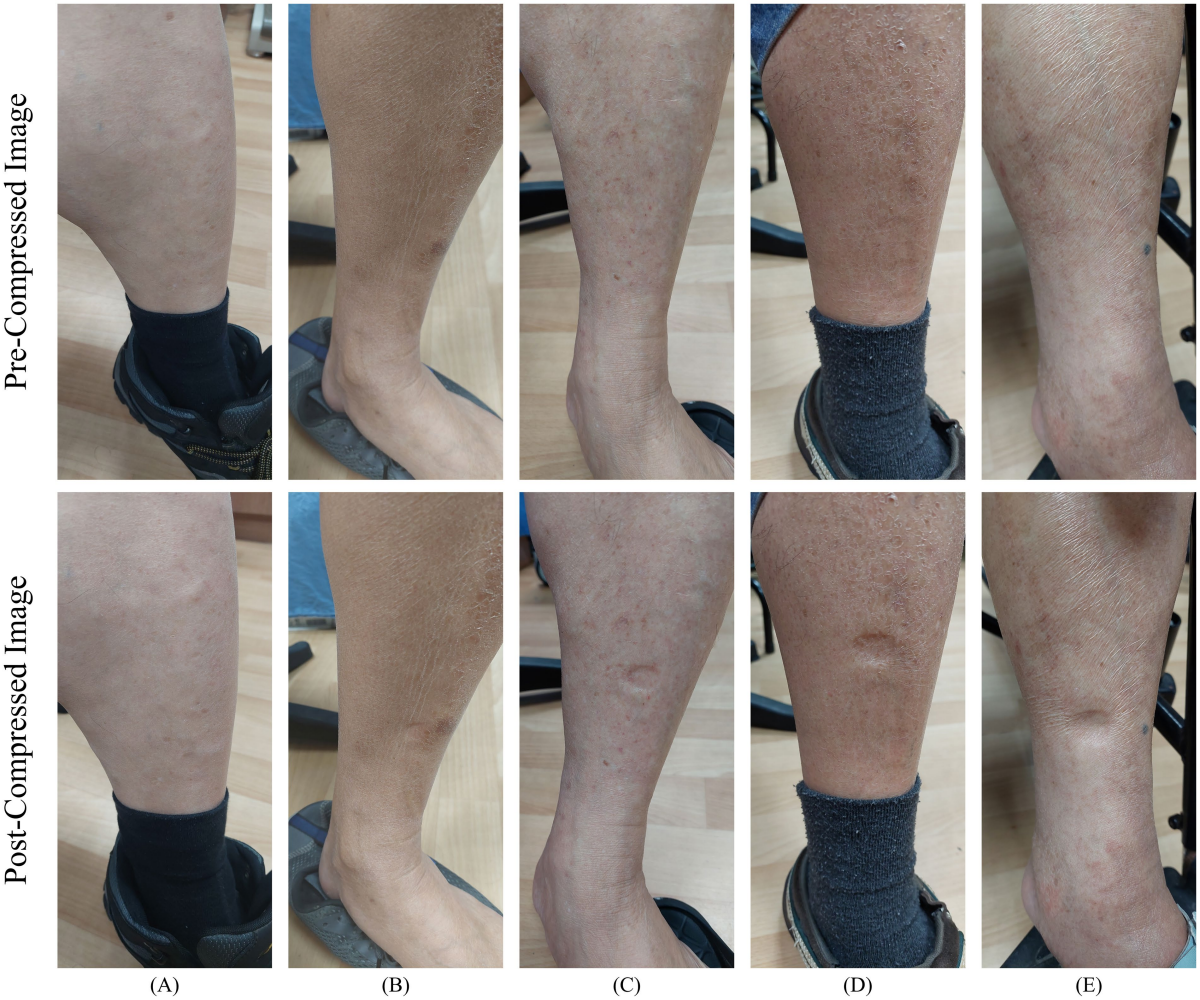
Examples of lower limb edema grading images. **(A)** Normal; **(B)** Edema $\;1^{+}$ ; **(C)** Edema $\;2^{+}$ ; **(D)** Edema $\;3^{+}$ ; **(E)** Edema $\;4^{+}$ .

The lower limb edema image dataset used in this study (Institutional Review Board [IRB]: 202500404B0) comprised five grading categories: normal, Edema $\;1^{+}$ , Edema $\;2^{+}$ , Edema $\;3^{+}$ , and Edema $\;4^{+}$ . A total of 1,011 paired images were collected, as illustrated in Figure 2, with each pair consisting of one pre-compressed and one post-compressed image. During routine follow-up visits, physicians regularly capture images based on the patient’s condition. All images were acquired using mobile phone cameras with a focus on the calf muscle regions and included both the left and right lower limbs in the pre- and post-compressed states.

Following image collection, physicians repeatedly reviewed and annotated the corresponding edema grades, which served as the ground truth for subsequent model training, detection, and classification. Upon completion of the annotation processes, the dataset was categorized and statistically summarized according to the different edema grades; the distribution of image quantities is presented in Table 2. All images used in this study were de-identified to remove personally identifiable information, thereby ensuring compliance with the IRB ethical standards and data protection regulations.

**Table 2:** Lower limb edema image dataset.

Category	Count (pairs)	Percentage
Normal	134	13%
Edema $\;1^{+}$	488	48%
Edema $\;2^{+}$	322	32%
Edema $\;3^{+}$	49	5%
Edema $\;4^{+}$	18	2%
Total	1,011	100%

Before model training, a preprocessing pipeline was applied to the images of the lower limb edema. The procedures primarily included automatic background removal, cropping, and rotation of partial images. The process aims to eliminate irrelevant information, focus on the target regions of interest, and enhance balance conditions through data augmentation, thereby improving the model’s ability to learn discriminative features.

**Figure 3:**
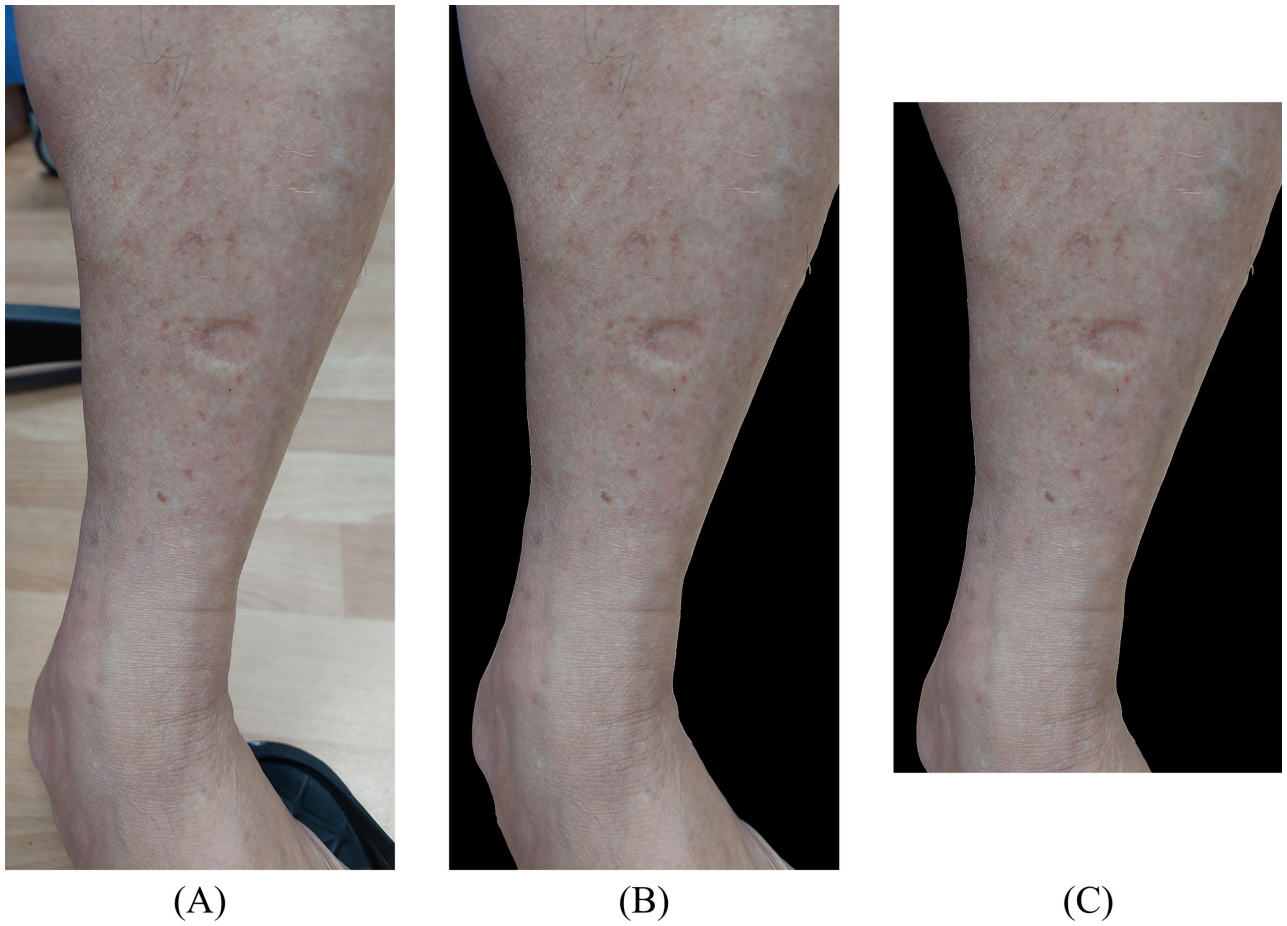
Illustration of data preprocessing. **(A)** Original image; **(B)** background removal; **(C)** cropped image (top and bottom 1/9 regions removed).

Since edema indentations were localized to the lower limb region, with all other areas considered background, background removal was performed on the original images to enhance the accuracy of feature extraction and learning. By eliminating irrelevant background information and retaining only the regions associated with edema indentations, as illustrated in Figures 3A, B, the subsequent model training processes achieved improved accuracy for automatic recognition.

Although background removal eliminated most non-target regions, the edema indentation area still occupied a relatively small proportion of the entire image and could be affected by external factors, such as the background of the foot or footwear. To further enhance the model’s focus on the target region, additional cropping was applied to the background-removed images. Specifically, one-ninth of the image was cropped from both the upper and lower edges to exclude potential nonessential information, as illustrated in Figure 3C.

Finally, to address the issue of class imbalance, data augmentation was performed during training by applying random rotation to images belonging to underrepresented categories (classes 3^+^ and 4^+^). This approach helped balance the distribution of training samples across all edema classes. In addition, an original image exhibited substantial variability in lighting conditions, shooting angles, capture distances, and skin texture. This diversity allowed the model to encounter the types of image quality variations commonly observed in clinical settings during training procedures, thereby enhancing its robustness to noise and environmental differences.

### 2.3 Detection module

For the first and second stages, a lower limb edema detection module was employed, which integrated contrast enhancement with image-recognition techniques to improve the accuracy and robustness of indentation detection. This module comprises three main technical processes: edema image detection, enhancement, and confirmation.

Edema image detection was performed using three YOLO-based models, namely YOLOv9, YOLOv10, and YOLOv11, to automatically localize the indentation regions associated with lower limb edema. Each model was trained and tested separately, and its detection performance was systematically evaluated to compare its effectiveness across different architectures.

**Figure 4:**
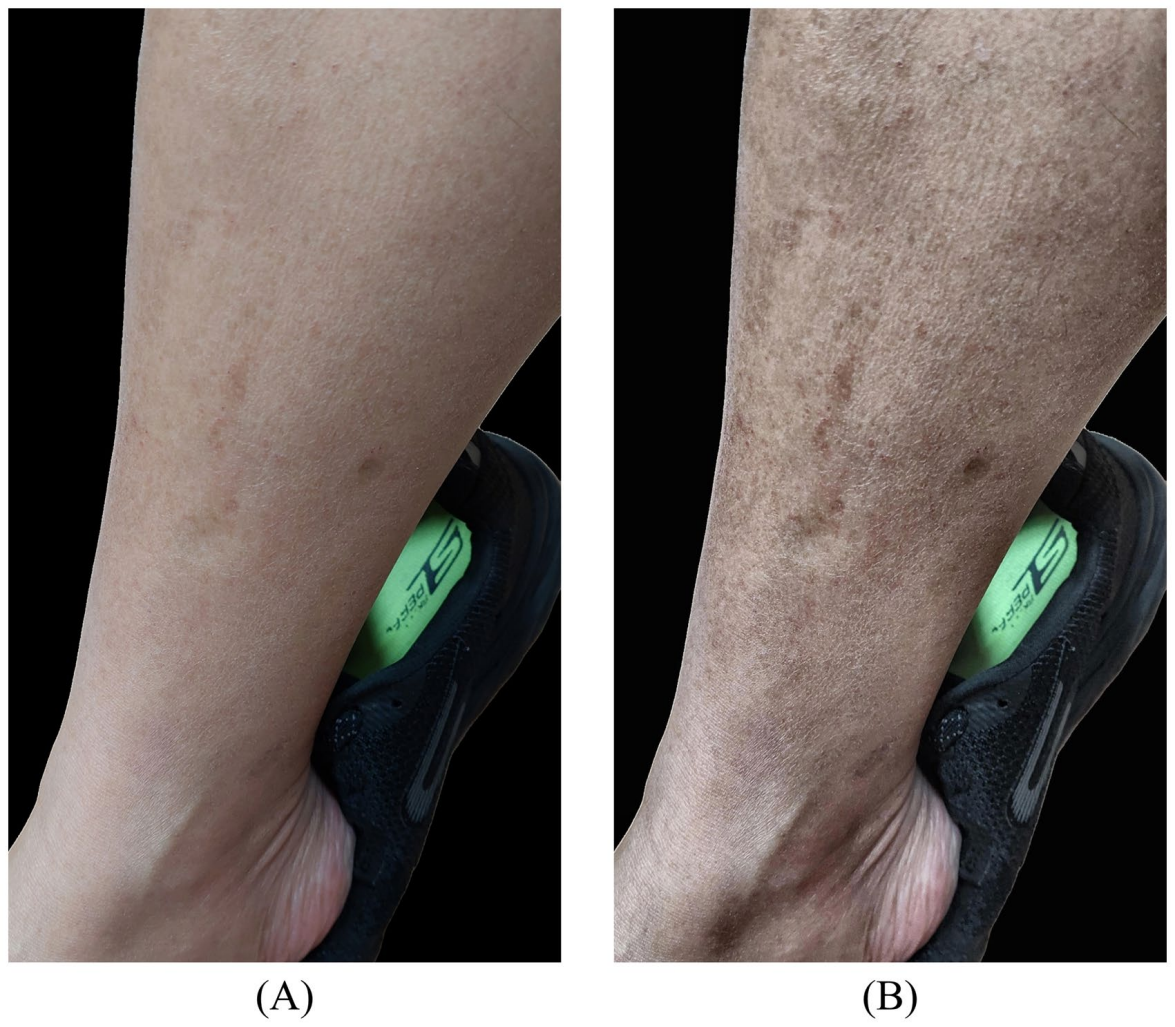
Illustration of CLAHE contrast enhancement: **(A)** background-removed original image; **(B)** contrast-enhanced image result.

Edema image enhancement was applied to cases where indentation features were less apparent, often due to uneven lighting, insufficient contrast, or variations in skin tone, which could adversely affect detection accuracy. To address this, contrast enhancement techniques, including HE, AHE, and CLAHE, were introduced prior to the second-stage model training, as illustrated in Figure 4. These methods were employed to improve detection performance, and their respective impacts on model accuracy were systematically analyzed.

**Figure 5:**
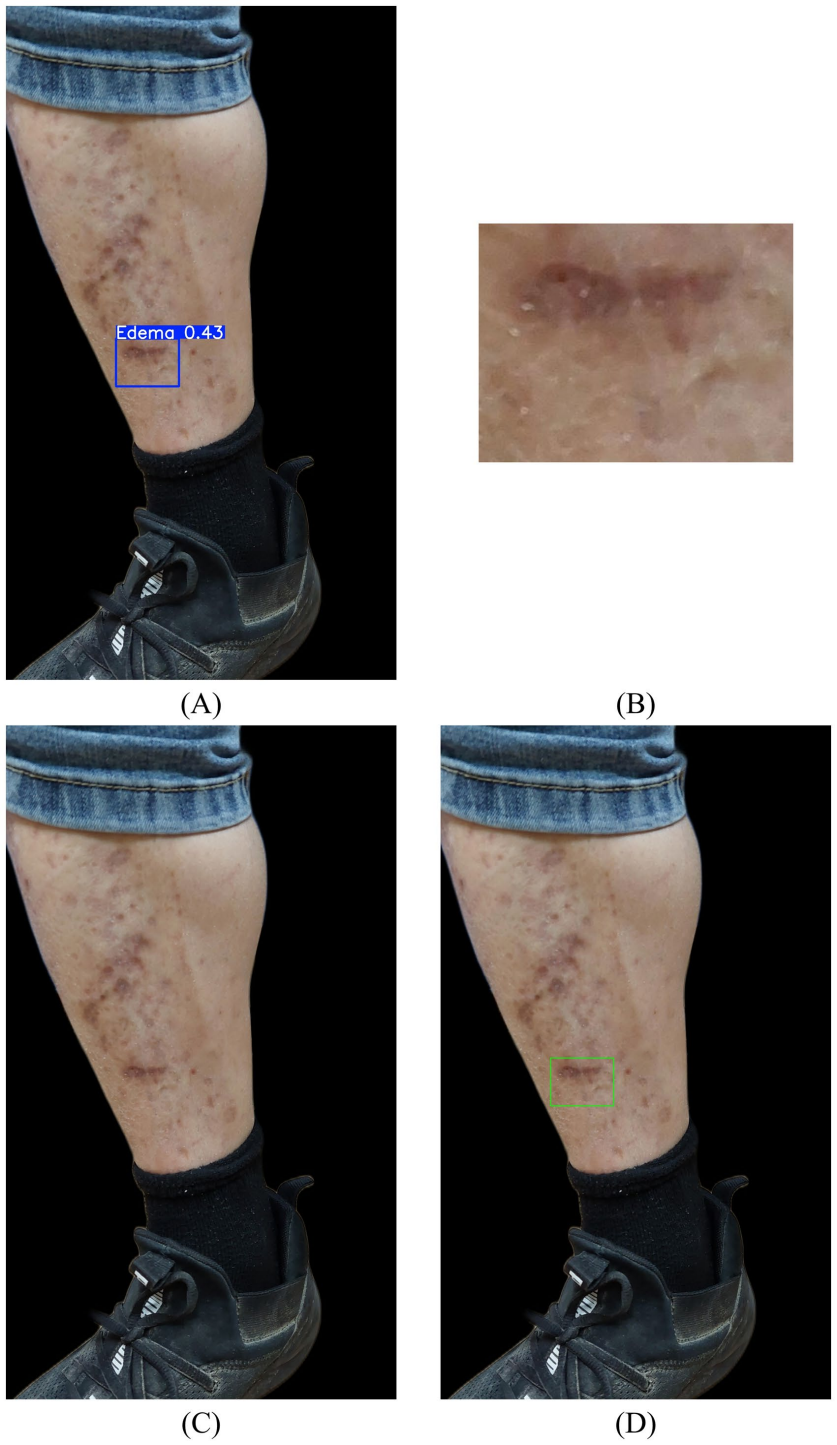
Examples of images successfully recovered through second-stage advanced edema confirmation analysis: **(A)** detection results after compressed image; **(B)** template matching; **(C)** before compressed image; **(D)** example of edema image confirmation.

Edema image confirmation incorporated the template-matching technique from OpenCV to validate the detection outcomes. In cases where non-edematous images were incorrectly classified as edema-positive in the first stage, the indentation region detected from the after-compression image was extracted as a matching template and compared with its corresponding before-compression image. This process confirms whether the predicted bounding box corresponds to the actual edema features, as illustrated in Figure 5.

The matching process began by extracting the predicted bounding box from the post-compressed image as a template, which was then applied using OpenCV’s MatchTemplate function. The corresponding region and its surrounding 3 × 3 grid in the pre-compressed image were searched for potential matches. Normalized squared differences were employed as a similarity metric, where a value of 0 indicated a perfect match, and values approaching 1 indicated poorer matches. In this study, the similarity threshold was set to 0.03 through several experiments; matches with similarity values below this threshold were considered valid detections, whereas those exceeding the threshold value were discarded. This matching procedure effectively improved the overall accuracy of edema detection.

### 2.4 Classification module

From the third to the fifth stage, a comprehensive confirmation process was employed for the lower limb edema classification module. These modules incorporate four classification models, VGG16, ResNet50, YOLOv8, and YOLOv11, to classify the images of indentation regions into their respective edema grades. Each model was trained and tested independently, and its classification performances were systematically evaluated and compared.

### 2.5 Implementation details

The experimental environment was established using the Windows 11 operating system equipped with an NVIDIA GeForce RTX 3090 GPU and 16 GB of memory. During the development of the deep learning models, a virtual environment was created using Anaconda, with Python as the primary programming language, and OpenCV was utilized for image processing. For model training, open-source frameworks including YOLO, PyTorch, and TensorFlow were employed to construct, train, and evaluate the predictive models. For the YOLO models, the parameter settings included the Stochastic Gradient Descent optimizer, learning rate of 0.01, batch size of eight, and 500 training epochs. The input images were resized to 640 × 640 pixels in RGB format. For the image classification models, input images were set to 224 × 224 pixels in RGB format, with CLAHE parameters configured at a clip limit of 2.0 and grid size of 8 × 8. To evaluate the predictive performance of the models on lower-limb edema images, multiple classification metrics were employed, including accuracy, precision, recall (also known as sensitivity), specificity, and F1-score. Recognition results are presented in the following sections.

## 3 Results

### 3.1 Performance of the first-stage preliminary edema detection

For the first-stage experiment, the image data were categorized into two classes, normal and edema conditions, with precise recognition from grades $1^{+}$ to $4^{+}$ . A total of 1,011 post-compressed images were obtained. To address the class imbalance, data augmentation was applied by randomly rotating the normal images, and the model training and testing were performed using five-fold cross-validation on the image dataset.

Three models, YOLOv9-c, YOLOv10-l, and YOLOv11-l, were trained and evaluated for edema detection. The confidence threshold was determined based on the confidence score corresponding to the highest average F1-score obtained during cross-validation. Finally, confusion matrices from all five cross-validation folds were aggregated to comprehensively assess the overall performance of each model for edema detection.

**Figure 6:**
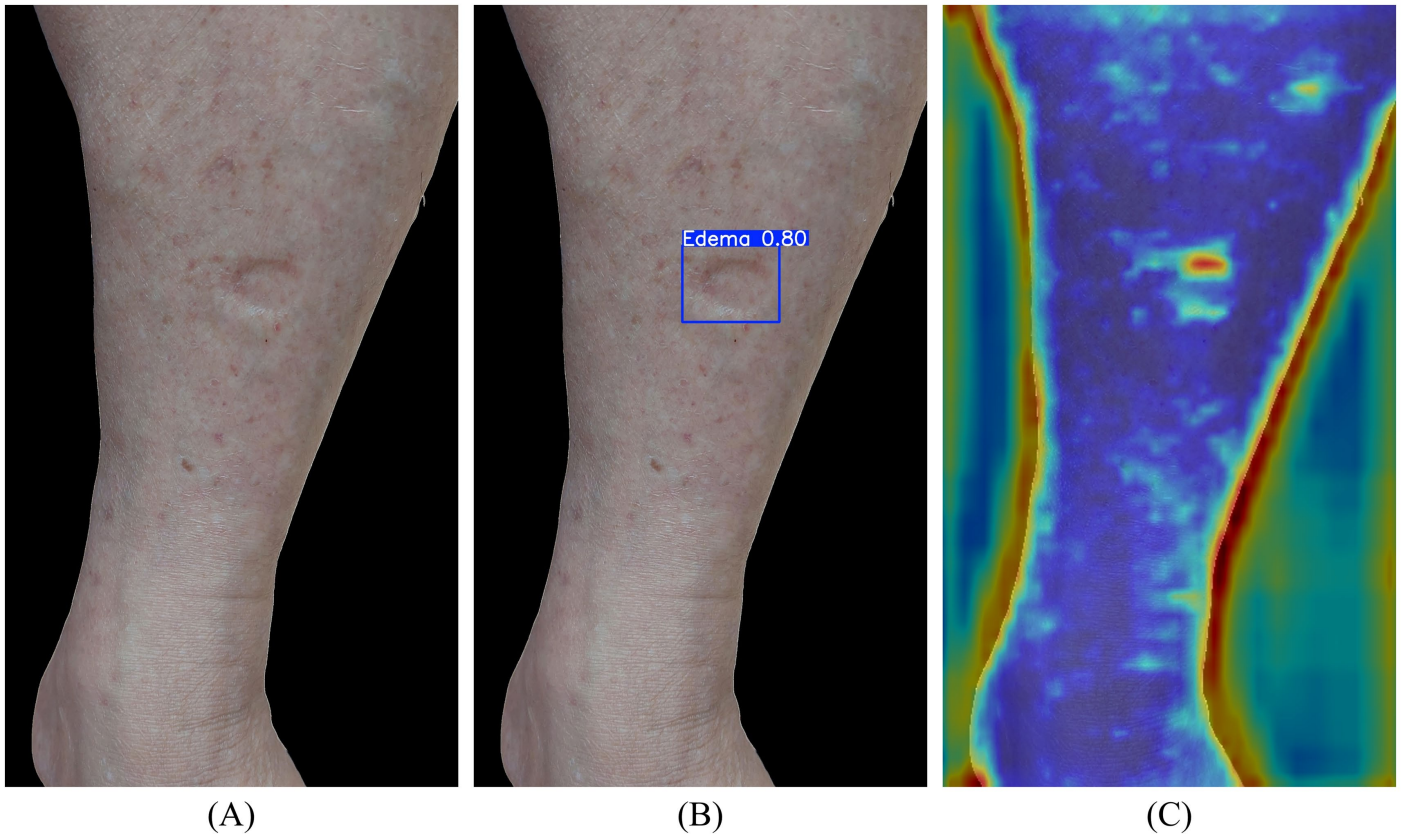
Detection results and their heatmaps. **(A)** Background-removed original image; **(B)** the first-stage detection results; **(C)** heat maps.

In the first stage, 1,011 post-compressed images were analyzed. The experimental results demonstrated that the models could effectively distinguish the presence of edema in the images. Among the tested models, YOLOv9-c achieved the best overall performance, with accuracies of 0.90, precision of 0.98, recall of 0.90, specificity of 0.87, and an F1-score of 0.94, as summarized in Table 3. Representative results of lower limb edema detection are shown in Figure 6. Although YOLOv10-l and YOLOv11-l exhibited competitive results for certain metrics, their overall performances were slightly inferior to that of YOLOv9-c. The superior performance of YOLOv9-c may be attributed to its incorporation into the Programmable Gradient Information (PGI) mechanism and the General Efficient Layer Aggregation Network (GELAN) convolutional architecture, which enhances the model’s ability to learn indentation-related features in complex lower limb edema images.

**Table 3:** Training results of various models in the first-stage preliminary edema detection.

Model	Accuracy	Precision	Recall (sensitivity)	Specificity	F1-score
YOLOv9-c	0.90 ± 0.02	0.98 ± 0.01	0.90 ± 0.02	0.87 ± 0.07	0.94 ± 0.02
YOLOv10-l	0.86 ± 0.02	0.99 ± 0.01	0.85 ± 0.03	0.94 ± 0.05	0.91 ± 0.02
YOLOv11-l	0.86 ± 0.03	0.98 ± 0.01	0.86 ± 0.05	0.87 ± 0.07	0.92 ± 0.02

### 3.2 Performance of the second-stage advanced contrast enhancement and edema confirmation

The second-stage experiment focused on reevaluating 204 lower limb edema images initially classified as non-edematous during the first-stage processes. To enhance feature visibility, three different contrast enhancement techniques were applied to these images, including HE, AHE, and CLAHE. The enhanced images were subsequently sent to train the YOLOv9-c model individually to identify edema cases that were overlooked during the first-stage detection.

Based on the results of the first-stage YOLOv9-c model, the lower-limb images initially classified as non-edematous were categorized as either normal or edematous. Edema refers to post-compressed images with edema annotations that were not detected in the first stage. In this phase, 204 post-compressed images were applied. Data augmentation was performed through random image rotation for both the normal and edema categories, and five-fold cross-validation was employed for model training and testing.

The YOLOv9-c model was the best-performing model in the first stage and was adopted for training and evaluation in this experiment. The confidence threshold was determined according to the confidence score corresponding to the optimal average F1-score obtained during cross-validation. Finally, the confusion matrices generated from the five cross-validation testing sets were aggregated to comprehensively evaluate the overall performance following contrast enhancement.

At this stage, advanced contrast enhancement and edema confirmation were applied to further determine the presence of the identified features, thereby improving the detection accuracy. Among the methods evaluated, CLAHE in combination with the YOLOv9-c model demonstrated the best performance, successfully recovering 31 missed edema cases while producing only 10 false-positive cases.

By comparison, HE and AHE resulted in a higher number of false detections. Superior CLAHE performance was selected to enhance the image contrast amplification locally, thereby improving the ability of the model to detect subtle indentation features in ambiguous edema images. Although HE increases the global contrast, its effectiveness is limited to images with fine structural details, highlighting the advantages of the CLAHE approach.

To further improve the detection accuracy, the indentation regions identified in the post-compressed images by the YOLOv9-c model during Stage 1 were compared with their corresponding before-compressed images using OpenCV’s template matching technique. This approach verified whether the predicted bounding boxes truly corresponded to edema indentations. Using this confirmation process, 11 misclassified images were successfully obtained.

Finally, the confusion matrix results obtained from the CLAHE-enhanced images were combined with the outcomes of the advanced edema confirmation processes to comprehensively evaluate the performance. The cumulative confusion matrix generated from the CLAHE-enhanced images was integrated with the results of advanced edema confirmation using template matching. This combined evaluation demonstrated the overall effectiveness of contrast enhancement and reconfirmation in edema detection, achieving an accuracy of 0.93, precision of 0.98, recall of 0.94, specificity of 0.88, and an F1-score of 0.96.

### 3.3 Performance of the third-stage mild and severe edema grading

In the third stage, images with confirmed edema status were extracted and classified into two groups according to severity: mild edema (Edema $\;1^{+}2^{+}$ ) and severe edema (Edema $\;3^{+}4^{+}$ ). A total of 821 post-compressed images were obtained. To address class imbalance, random rotations were applied for data augmentation of the Edema $\;3^{+}4^{+}$ images, and five-fold cross-validation was conducted for model training and testing.

Four classification models–VGG16, ResNet50, YOLOv8-l-cls, and YOLOv11-l-cls–were trained and evaluated simultaneously at this stage. The confusion matrices generated from the five cross-validation folds were aggregated to compare the overall classification performance of each model.

In the third stage, with 821 post-compressed images, the experimental results indicated that YOLOv11-l-cls achieved the most effective classification performance, with an accuracy of 0.98, precision of 0.97, recall of 0.92, and F1-score of 0.94, as presented in Table 4. The superior performance of YOLOv11-l-cls may be due to its advanced convolutional architecture, which enhances the model’s ability to capture and learn edema-related features.

**Table 4:** 5-fold training results of various models for third-stage mild and severe edema grading. $(1^{+}2^{+}$ vs. $3^{+}4^{+}).$

Model	Accuracy	Precision	Recall	F1-score
VGG16	0.95 ± 0.02	0.84 ± 0.08	0.84 ± 0.11	0.84 ± 0.08
ResNet50	0.96 ± 0.02	0.86 ± 0.05	0.88 ± 0.09	0.87 ± 0.05
YOLOv8-l-cls	0.98 ± 0.01	0.97 ± 0.04	0.89 ± 0.03	0.92 ± 0.03
YOLOv11-l-cls	0.98 ± 0.01	0.97 ± 0.03	0.92 ± 0.04	0.94 ± 0.02

### 3.4 Performance of the fourth-stage advanced analysis of mild edema grading

This stage was based on the superior classification performance obtained in stage 3, where the images were categorized as Edema $\;1^{+}$ and Edema $\;2^{+}$ . Specifically, this stage focused on separating the mild edema grading conditions. In total, 751 post-compressed images were included. To address class imbalance, random rotation processes were applied for the data augmentation of Edema $\;2^{+}$ images, and five-fold cross-validation was employed for model training and testing.

Four classification models–VGG16, ResNet50, YOLOv8-l-cls, and YOLOv11-l-cls–were trained and evaluated for comparison. The confusion matrices generated from the five cross-validation folds were aggregated to comprehensively assess the overall classification performance of each model.

For the advanced analysis of mild edema grading involving 751 post-compressed images, the experimental results showed that YOLOv11-l-cls achieved the most effective classification outcomes, with an accuracy of 0.94, precision of 0.94, recall of 0.94, and F1-score of 0.94, as summarized in Table 5.

**Table 5:** 5-fold training results of various models for fourth-stage advanced analysis of mild edema grading $(1^{+}$ vs. $2^{+}).$

Model	Accuracy	Precision	Recall	F1-score
VGG16	0.85 ± 0.02	0.85 ± 0.02	0.84 ± 0.02	0.85 ± 0.02
ResNet50	0.86 ± 0.02	0.86 ± 0.02	0.86 ± 0.02	0.86 ± 0.02
YOLOv8-l-cls	0.93 ± 0.04	0.92 ± 0.04	0.92 ± 0.04	0.92 ± 0.04
YOLOv11-l-cls	0.94 ± 0.02	0.94 ± 0.02	0.94 ± 0.03	0.94 ± 0.02

### 3.5 Performance of the fifth-stage advanced analysis of severe edema grading

The last stage was based on the superior classification outcomes obtained in stage 3 for severe edema grading, where the images were categorized as either Edema $\;3^{+}$ or Edema $\;4^{+}$ for further advanced analysis. A total of 56 post-compressed images were obtained. To address class imbalance, data augmentation through random rotation was applied to both the Edema $\;3^{+}$ and Edema $\;4^{+}$ samples, and five-fold cross-validation was employed for model training and testing.

Four classification models, namely VGG16, ResNet50, YOLOv8-l-cls, and YOLOv11-l-cls, were applied again for the training and evaluation processes. The confusion matrices generated from the five cross-validation folds were aggregated to comprehensively evaluate the overall classification performance.

For the fifth stage, 56 after-compressed images were reconfirmed, and the results showed that YOLOv11-l-cls achieved the most effective classification performance with an accuracy of 0.93, precision of 0.93, recall of 0.90, and F1-score of 0.92, as presented in Table 6.

**Table 6:** 5-fold training results of various models for fifth-stage advanced analysis of severe edema grading $(3^{+}$ vs. $4^{+}).$

Model	Accuracy	Precision	Recall	F1-score
VGG16	0.75 ± 0.13	0.71 ± 0.19	0.70 ± 0.11	0.70 ± 0.13
ResNet50	0.77 ± 0.08	0.73 ± 0.12	0.73 ± 0.07	0.73 ± 0.07
YOLOv8-l-cls	0.91 ± 0.01	0.92 ± 0.02	0.88 ± 0.06	0.89 ± 0.02
YOLOv11-l-cls	0.93 ± 0.05	0.93 ± 0.04	0.90 ± 0.09	0.92 ± 0.07

### 3.6 Performance comparison with mainstream models

To compare classification strategies, an integrative analysis was conducted based on the best-performing detection and classification models at each stage. When the system was configured for three-class automatic recognition (Normal $/1^{+}2^{+}/3^{+}4^{+}$ ), the average accuracy could reach 0.91. However, when the system was expanded to four-class recognition (Normal $/1^{+}/2^{+}/3^{+}4^{+}$ ), the average accuracy decreased by 4%, dropping to 0.87. For five-class recognition (Normal $/1^{+}/2^{+}/3^{+}/4^{+}$ ), the average accuracy remained at 0.87, but the F1-score achieved 0.84 and decreased slightly by 2%. Table 7 further summarized the detailed performance metrics, including accuracy precision, recall, and F1-score, for all five edema classes. These results suggest that as the number of classification levels increases, the feature differences between adjacent edema grades become ambiguous, thereby increasing the risk of misclassification.

**Table 7:** Class-wise performance metrics.

Class-wise performance metrics	Accuracy	Precision	Recall	F1-score
Three-class classification (Normal $/1^{+}2^{+}/3^{+}4^{+}$ )	0.92	0.86	0.88	0.87
Four-class classification (Normal $/1^{+}/2^{+}/3^{+}4^{+}$ )	0.87	0.86	0.87	0.86
Five-class classification (Normal $/1^{+}/2^{+}/3^{+}/4^{+}$ )	0.87	0.86	0.85	0.84

## 4 Discussion

**Figure 7:**
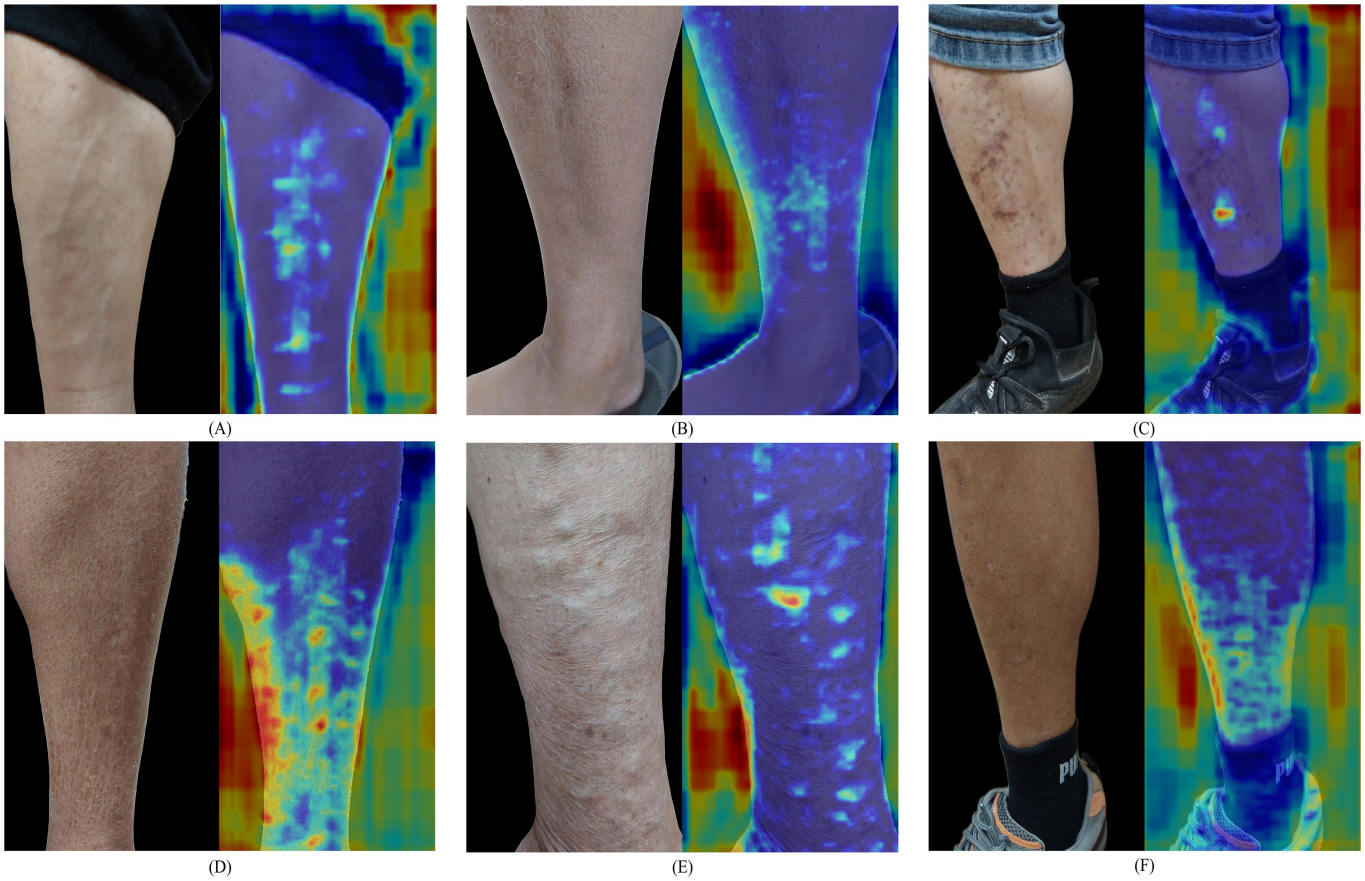
Failure case examples: **(A)** image acquisition angle and resolution; **(B)** high similarity of edema indentation features; **(C)** presence of wounds or scars on the skin; **(D)** complex skin texture; **(E)** multiple indentation-related skin conditions; **(F)** variations in skin pigmentation.

To further investigate the potential reasons for model misclassification, representative cases of incorrect predictions were selected for detailed analysis. These misclassified cases, illustrated in Figure 7, were examined to explore the possible reasons for the recognition errors. A comprehensive analysis revealed several key factors influencing model accuracy, including imaging conditions, characteristics of the skin surface, and inherent visual features of edema.

### 4.1 The detailed misclassified factors were summarized as follows

(A) Image acquisition angle and resolution: When the compression site is misaligned, the camera angle position is inappropriate, the resolution is insufficient, and the indentation features become indistinct, thereby decreasing the model’s prediction ability.
(B) High similarity of edema indentation features: the visual characteristics of certain edema grades were highly similar, making it difficult for the model to distinguish between them and constituting a primary cause of misclassification.
(C) Presence of wounds or scars on the skin: Prominent wounds or scars on the skin may mislead the prediction model and result in incorrect predictions.
(D) Complex skin texture: Factors such as pronounced wrinkles, fissures, tattoos, or protruding veins may interfere with the recognition of indentation features, thereby decreasing the prediction model performance.
(E) Multiple indentation-related skin conditions: Irregular depressions caused by nearby skin diseases or non-edema conditions may be confused with edema-related indentations, thus reducing prediction accuracy.
(F) Variations in skin pigmentation: Darker skin tones or significant color differences around the compression area may hinder accurate feature recognition under uneven lighting or insufficient contrast.
(G) Timing of post-compressed imaging: The timing of image acquisition after compression directly affects indentation visibility. If the image is obtained after a long time, the indentation features would gradually disappear as the skin rebounds, thereby decreasing the recognition accuracy.

Considering the aforementioned factors, the proposed model might be influenced by non-edema-related features when processing real-world clinical images, indicating that relying on a single image for judgment still contains inherent limitations. First, misclassifications were caused by poor image quality, including inconsistent acquisition angles, uneven illumination, and blurred images, which might weaken diagnostically relevant skin indentation or texture features and make it difficult for the model to extract stable and discriminative features from the input images. Such errors were more likely related to image quality limitations, as an example illustrated in Figure 7A.

In addition, in grade 1^+^ cases, the edema indentations were sometimes with subtle differences, and the prediction model might be failed due to only captured with fine-grained differences (shown in Figure 7B). It identified features exhibiting high visual similarities. Furthermore, when identified edema indentations, the model might classify existed wounds or scars with similar appearances as edema regions in a wrong way. However, an image-matching technique was also additionally employed to correct such conditions, and it was demonstrated in Figure 7C.

Finally, when handling complex skin textures, the proposed model was still constrained by the high intrinsic variability of skin structures. This limitation would affect feature extraction and recognition processes, particularly in a region with complex texture or multiple localized indentations. A few representative examples were shown in Figures 7D–F, indicating that the prediction model for various feature representation could be improved through more different cases collection. To preserve the authenticity of the clinical images and to enhance the generalizability of the proposed model, such challenging cases were retained in both training and testing stages. This approach allowed the proposed system to reflect a comprehensive evaluation of our model performance and to confront practical difficulties and limitations encountered in real-world clinical data.

## 5 Conclusion

The experimental results demonstrate that the proposed system achieves automatic recognition across different classification strategies, with an average accuracy ranging from 87 to 93%. Nevertheless, discrepancies remained between the real lower limb edema images and the simulated skin models. These differences include broader image capture ranges, which may increase the likelihood of misclassification, complex and heterogeneous skin surface textures and pigmentation, and variations in background environments, all of which could introduce confusion into the model.

The annotation of edema images poses particular challenges, primarily owing to subtle differences in indentation depth among various edema grades, which are often difficult to discern with the naked eye. Moreover, the high similarity of certain indentation patterns further constrains the model’s recognition performance, thereby necessitating a collaborative discussion and verification among multiple physicians.

Despite these challenges, the grading strategy employed in this study yielded one of the most accurate performances reported to date for the automatic detection and grading classification of lower-limb edema, validating both the effectiveness and feasibility of the proposed system. The findings of this study can be applied to clinical decision support and home-based self-monitoring systems. Such applications could enhance the reliability of edema grading for medical professionals and facilitate rapid self-assessment of patients in remote or underserved areas.

### 5.1 Future prospects

This study proposes an automatic detection and grading classification system for lower limb edema, with experimental results demonstrating high recognition performance and promising potential for clinical applications. Nonetheless, several technical challenges and areas of improvement remain before translation into routine clinical practice. Although approximately 1,000 images were collected, the limited number of cases in the Edema 3^+^ and 4^+^ categories constrained the model’s ability to accurately recognize severe edema. Future work should focus on expanding the dataset to include samples from multiple medical institutions, diverse populations, and various age groups, thereby enhancing both data diversity and the accuracy and robustness of the model. Furthermore, it is recommended to incorporate Gaussian noise into image rotation processes and employ Generative Adversarial Networks (25) to generate synthetic edema images, thereby enriching the dataset and alleviating the problem of class imbalance.

Moreover, the current model was limited by its reliance on single static images. Future work may incorporate dynamic recordings of the indentation rebound time, enabling the model to capture the temporal characteristics of edema, thereby achieving precise detection and grading. Regarding model design, future research could also consider integrating the strengths of different network architectures, along with optimizing the model structure and hyperparameters, to further enhance detection and classification accuracy. Overall, although this study demonstrated a promising performance and preliminary clinical value, opportunities for advancement remain in data expansion, model integration, and system implementation. Continued refinement of these aspects may ultimately lead to the development of a more precise and robust system for the automatic detection and grading of lower-limb edema.

## Data Availability

The datasets presented in this article are not readily available because the right of the image dataset belong to patients. Requests to access the datasets should be directed to Tun-Wen Pai, twp@ntut.edu.tw.

## References

[1] Chu EC Wong JT. Subsiding of dependent oedema following chiropractic adjustment for discogenic sciatica. Eur J Mol Clin Med. (2018) 5:12–5. doi: 10.5334/ejmcm.250

[2] Hogan M. Medical-surgical nursing. 2nd ed. London, United Kingdom: Pearson Education, Limited (2007).

[3] Brodovicz KG. Reliability and feasibility of methods to quantitatively assess peripheral edema. Clin Med Res. (2009) 7:21–31. doi: 10.3121/cmr.2009.819, 19251582 PMC2705274

[4] Menegatti E Tessari M Gianesini S. "Clinical examination in lower limb edema" In: Tiwary SK editor. Approach to lower limb oedema. New York, USA: Springer (2022) 55–63.

[5] Seidel HM Stewart RW Ball JW Dains JE Flynn JA Solomon BS. Mosby’s guide to physical examination-E-book. St. Louis, Missouri: Elsevier Health Sciences (2010).

[6] O. Company. SurroSense Rx (2013). Available online at: https://www.mobihealthnews.com/news/surrosense-rx-foot-sensor-hits-market-aims-prevent-diabetic-ulcers (accessed November 13, 2013)

[7] S.-C. Company. Siren socks (2018). Available online at: https://siren.care/ (accessed November 10, 2020)

[8] Yahathugoda C Weiler MJ Rao R De Silva L Dixon JB Weerasooryia MV . Use of a novel portable three-dimensional imaging system to measure limb volume and circumference in patients with filarial lymphedema. Am J Trop Med Hyg. (2017) 97:1836–42. doi: 10.4269/ajtmh.17-0504, 29141750 PMC5805069

[9] George S. M. Langley B. D. Weaver E. M. B. Hardin S. R. Jianchu Y. (2016). Design of peripheral edema measurement device for home use. 2016 38th annual international conference of the IEEE engineering in medicine and biology society (EMBC), 4387–4390. 10.1109/EMBC.2016.7591699 28269250

[10] Williams K. Han M. Hardin S. George S. Yao J. (2018). AERO: an objective peripheral edema measurement device. 2018 40th annual international conference of the IEEE engineering in medicine and biology society (EMBC), 5914–5917. 10.1109/EMBC.2018.8513657 30441682

[11] Chen J. Mao T. (2018). Camera-based peripheral edema measurement using machine learning. 2018 IEEE international conference on healthcare informatics (ICHI), 457–458.

[12] Smith AG Perez R Thomas A Stewart S Samiei A Bangalore A . Objective determination of peripheral edema in heart failure patients using short-wave infrared molecular chemical imaging. J Biomed Opt. (2021) 26:105002. doi: 10.1117/1.JBO.26.10.105002, 34689443 PMC8541742

[13] Sheng JQ Hu PJ-H Liu X Huang T-S Chen YH. Predictive analytics for care and management of patients with acute diseases: deep learning–based method to predict crucial complication phenotypes. J Med Internet Res. (2021) 23:e18372. doi: 10.2196/18372, 33576744 PMC7910123

[14] Kermany DS Goldbaum M Cai W Valentim CC Liang H Baxter SL . Identifying medical diagnoses and treatable diseases by image-based deep learning. Cell. (2018) 172:1122–1131.e9. doi: 10.1016/j.cell.2018.02.010, 29474911

[15] Ker J Wang L Rao J Lim T. Deep learning applications in medical image analysis. IEEE Access. (2017) 6:9375–89. doi: 10.1109/ACCESS.2017.2788044

[16] Yang TH Sun YN Li RS Horng MH. The detection and classification of scaphoid fractures in radiograph by using a convolutional neural network. Diagnostics. (2024) 14:2425. doi: 10.3390/diagnostics14212425, 39518391 PMC11545356

[17] Tarimo SA Jang MA Ngasa EE Shin HB Shin H Woo J. WBC YOLO-ViT: 2 way—2 stage white blood cell detection and classification with a combination of YOLOv5 and vision transformer. Comput Biol Med. (2024) 169:107875. doi: 10.1016/j.compbiomed.2023.107875, 38154163

[18] Elazab N Gab-Allah WA Elmogy M. A multi-class brain tumor grading system based on histopathological images using a hybrid YOLO and RESNET networks. Sci Rep. (2024) 14:4584. doi: 10.1038/s41598-024-54864-6, 38403597 PMC10894864

[19] Wang C. -Y. Yeh I. -H. Liao H. -Y. M. YOLOv9: learning what you want to learn using Programmable Gradient Information. arXiv [Preprint]. arXiv:240213616 (2024).

[20] Wang A. Chen H. Liu L. Chen K. Lin Z. Han J. . YOLOv10: real-time end-to-end object detection. arXiv [Preprint]. arXiv:240514458 (2024).

[21] Jocher G. Ultralytics YOLOv11 (2024). Available online at: https://github.com/ultralytics/ultralytics (Accessed September 18, 2024).

[22] Simonyan K. Zisserman A. Very deep convolutional networks for large-scale image recognition. arXiv [Preprint]. arXiv:1409.1556 (2014).

[23] He K. Zhang X. Ren S. Sun J. (2016). Deep residual learning for image recognition. In Proceedings of the IEEE conference on computer vision and pattern recognition (CVPR), 770–778.

[24] Jocher G. Ultralytics YOLOv8 (2024). Available online at: https://github.com/autogyro/yolo-V8 (Accessed July 5, 2024).

[25] Goodfellow I. Pouget-Abadie J. Mirza M. Xu B. Warde-Farley D. Ozair S. . Generative Adversarial Networks. arXiv [Preprint]. arXiv:1406.2661 (2014).

